# The case of Schnitzler syndrome in one single rheumatologic center

**DOI:** 10.1186/1546-0096-13-S1-P164

**Published:** 2015-09-28

**Authors:** S Salugina, E Fedorov, V Gorodetskiy, M Evsikova, N Lopatina

**Affiliations:** 1 Nasonova Research Institute of Rheumatology, Pediatric Department, Moscow, Russian Federation; 2 Nasonova Research Institute of Rheumatology, Rheumatology Department, Moscow, Russia, Moscow, Russian Federation

## Background

Schnitzler syndrome is characterized by chronic, nonpruritic urticaria in association with recurrent fever, bone pain, arthralgia or arthritis, and a monoclonal immunoglobulin M (IgM) gammopathy. Pathogenesis of Schnitzler syndrome is unclear. Some hypothesize that the deposition of the IgM paraprotein, leading to the formation of immune complexes and the activation of the complement cascade, is responsible for the cutaneous manifestations. Another proposed mechanism involves the uncontrolled activation of interleukin 1-alpha (IL-1 α).

## Aim

To report the case of Schnitzler syndrome in our clinic, that to be considered like rare entities.

## Case report

A 44-year-old Caucasian man had had symptoms beginning at the age of 40 years, including fever, fatigue, recurrent urticaria, conjunctivitis, swelling of the eyelids, angioedema sometimes. Urticaria lasted 2-3 days and then disappeared without sequelae. These episodes recurred once per month. The laboratory findings included an ESR of up to 36 mm/h, neutrophil leukocytosis - 12.4-18.7 x 109, C-reactive proteine rise -107 mg/l (normal 0-5.0 mg/l), ferritin - 415 mkg/l (normal 20-150 mkg/l),, SAA-127 ng/ml (normal 0-6, 4 ng/ml), ANA and RF were negative, a monoclonal immunoglobulin M (IgM) gammopathy detected with serum immunoelectrophoresis in a concentration 5 g/L. A histopathologic examination was not done. The patient was treated with nonsteroidal anti-inflammatory drugs (NSAIDs), systemic steroids intravenously (120-90-60 mg) and orally (20 mg), methotrexate 20 mg/week, that were somewhat effective at controlling the urticaria and fever. Differential diagnoses was made with Systemic Lupus Erythematosus, Still's disease in adult, Acute Urticarial Vasculitis, also hereditary autoinflammatory diseases - cryopyrin-associated periodic syndromes (CAPS) - Muckle-Wells syndrome (MWS).

A genetic analysis on the patient did not show a mutation in the NLRP3 (CIAS1), MVK, TNFRSF1A genes.

## Conclusions

We report the rare case of Schnitzler syndrome. It was very difficult to diagnose and confirm this diagnosis. The clinical and laboratory signs are wery similar with MWS. But later then in pts with MWS adult age (40 years old) of onset of disease and also a monoclonal immunoglobulin M (IgM) gammopathy put the correct diagnosis.

## Consent to publish

Written informated consent for publication of their clinical details was obtained from the patient/parent/guardian/relative of the patient.

